# Quantifying drug tissue biodistribution by integrating high content screening with deep-learning analysis

**DOI:** 10.1038/s41598-020-71347-6

**Published:** 2020-09-01

**Authors:** Zhuyin Li, Youping Xiao, Jia Peng, Darren Locke, Derek Holmes, Lei Li, Shannon Hamilton, Erica Cook, Larnie Myer, Dana Vanderwall, Normand Cloutier, Akbar M. Siddiqui, Paul Whitehead, Richard Bishop, Lei Zhao, Mary Ellen Cvijic

**Affiliations:** 1 Lead Discovery and Optimization, Bristol-Myers Squibb, 3551 Lawrenceville Road, Princeton, NJ 08540 USA; 2 Information Technology for R&D, Bristol-Myers Squibb, Princeton, NJ USA; 3 Translational Medicine, Bristol-Myers Squibb, Princeton, NJ USA; 4 Immunoscience Biology Discovery, Bristol-Myers Squibb, Princeton, NJ USA; 5 Cardiovascular Translational Research, Bristol-Myers Squibb, Hopewell, NJ USA

**Keywords:** Imaging, Pharmacodynamics

## Abstract

Quantitatively determining in vivo achievable drug concentrations in targeted organs of animal models and subsequent target engagement confirmation is a challenge to drug discovery and translation due to lack of bioassay technologies that can discriminate drug binding with different mechanisms. We have developed a multiplexed and high-throughput method to quantify drug distribution in tissues by integrating high content screening (HCS) with U-Net based deep learning (DL) image analysis models. This technology combination allowed direct visualization and quantification of biologics drug binding in targeted tissues with cellular resolution, thus enabling biologists to objectively determine drug binding kinetics.

## Introduction

To achieve a desirable therapeutic response, a drug must first distribute to the intended tissue or organ, bind to the cell surface or intra-cellular proteins, and then induce a cascade of intracellular and/or intercellular reactions to modulate disease processes. Due to the complexity of the disease tissue microenvironment, as well as drug metabolism and clearance, in vitro drug-cell binding assays or ex vivo tissue lysate assays barely recapitulate in vivo drug distributions, thus impeding the development of predictable correlation models between drug dosing and therapeutic efficacy or adverse effects^1–5^. In vivo and ex vivo radiography or total fluorescent-based tissue imaging methods have been generally used to visualize and quantify drug distribution in tissues obtained from animal models; however, due to low resolution (millimeter scale), these technologies neither correlate drug binding with cell types of interest, nor differentiate specific vs. non-specific drug binding, making data interpretation complicated^6–9^.

The image resolution of multi-color confocal fluorescent microscopy is in the submicrometer scale and renders this technology an ideal tool for the study of drug distribution with cellular resolution on tissues collected from animal models. Furthermore, this technology would enable correlations of drug loading with therapeutic efficacy by co-staining with pharmacodynamics (PD) biomarkers. However, the throughput of traditional confocal fluorescent imaging is limited, and the development of image analysis algorithms is time consuming and requires considerable coding skill to develop software for each case. Therefore, multi-color confocal bioimaging technology has not become the main tool for quantifying drug distribution in animal models.

High content screening (HCS) or automated microscope-based screening technology^10^ has been broadly used to visualize drug binding in recombinant cell lines or primary cells with well-controlled cell density (thus controlled space between cells) in each well of a microtiter plate. Furthermore, a spectrum of nucleus-centric and automated image analysis tools have been developed and successfully applied for different biological processes, including quantification of drug binding^11^. However, these nucleus-centric, high-throughput image analysis methods lack the flexibility needed for complex tissues in which different cell types are tightly packed. Therefore, segmentation of nuclei in tissue using current HCS-based approaches is an insurmountable challenge.

Recent advances in deep learning (DL)-based image analysis provides a path forward to more effectively quantify drug biodistribution by multiplexed fluorescent imaging. Without coding, a DL model can automatically identify various tissue/cellular features by learning from annotated images, and then defining relevant structures^12–16^. Therefore, DL-based image analysis approach likely would allow biologists to accurately and objectively quantify drug distribution in specific tissues.

In this report, we detailed the applications of U-Net on quantifying drug biodistribution. U-Net is a Convolutional Neural Network (CNN)-based architecture developed to perform segmentation and detection tasks on microscopic images of cellular and tissue biosamples^12,16^. In addition to the conventional component (the encoder) of CNN that extracts various features from input images, U-Net has multiple layers of up-convolutions (the decoder) that increase spatial resolution at the succeeding layers. Consequently, the output images have the same spatial resolution as that of the input ones. Therefore, each pixel in the input images can be classified into different biological components by a U-Net model. This level of details allows one to determine drug binding in complex tissues with cellular resolution.

The present work provides a generic, high-throughput quantification method for biologics drug distribution in complex animal tissues by integrating HCS-based multi-color tissue imaging technology with the U-Net based imaging analysis algorithm.

## Results

### Adaptation of HCS for tissue image acquisition

**Figure 1:**
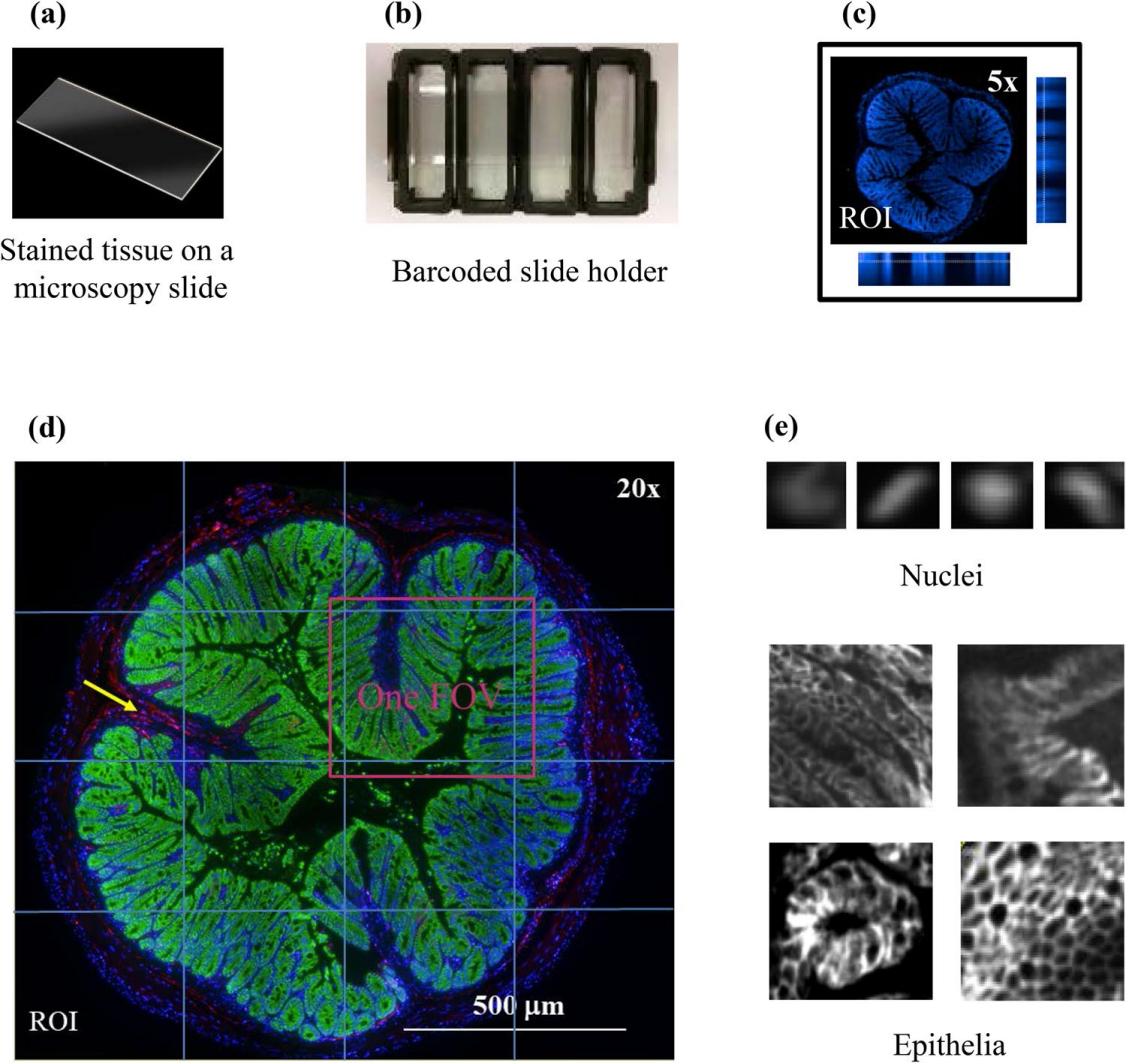
Automated tissue image acquisition using HCS. (**a**) A whole frozen colon tissue slice from a αCDH-A647 treated animal was mounted to a standard microscope slide and stained with anti-EpCAM and DAPI. (**b**) A slide holder with standard microtiter plate footprint was built to house 4 tissue slides in order to facilitate automated image acquisition using the PHENIX HCS reader. (c) A 5 × air objective lens was applied to localize all nuclei (blue), thus defining ROI and providing an optimal Z height starting point. Side images are orthogonal views of maximum nuclear intensity at different Z heights. (**d**) Automated multi-color (blue: nucleus; green: EpCAM; red: αCDH-A647) rescan on ROI guided by PRECISCAN was conducted using a 20 × water-immersion objective lens. Images from all FOVs were then montaged for analysis. Yellow arrow points to one example of an area where αCDH-A647 was trapped in the interstitial space. (**e**) Different features of nuclei (top) and epithelia (bottom).

Cadherin 17 is a protein mainly expressed in the gastrointestinal tract. Here, we use whole slice colon and small intestine tissues obtained from mice treated with Alexa 647 dye labeled anti-cadherin 17 antibody (αCDH-A647) as models to illustrate the utility of a generic drug quantification method. Frozen whole slice tissue samples from the gastrointestinal tract represents one type of complex disease tissue^17^. First, nuclear and epithelial cell structures are highly variable due to differences in cell cycle, location in the tissue, disease stage and orientation in the cut tissue slice (Fig. 1e). Second, drug is often trapped in interstitial spaces (Fig. 1d). Finally, large-sized frozen tissues often cannot adequately adhere to a microscope glass slide. The “wavy” tissue slides may result in blurry images.

Prior to image acquisition by PerkinElmer’s Opera PHENIX HCS reader, tissue nuclei were stained with DAPI and total lumen epithelial areas were marked by anti-EpCAM antibody tagged with Alexa 488 dye. Stained tissues were placed in a slide holder with standard microtiter plate footprint (Fig. 1a, b). To increase image acquisition throughput without sacrificing image quality, Harmony PRECISCAN, a PHENIX built-in pattern recognition image processing software for automated pre-scan and re-scan of regions-of-interest (ROI), was used to streamline the process. This software first performed a low magnification (air objective lens 5X, NA = 0.16) tissue topography survey (x, y & z locations) based on all nuclear stains to define ROI (Fig. 1c). Based on this preliminary survey, a second round of multi-color image acquisition on the ROI was performed at a higher magnification (water objective lens 20X, NA = 1.0) (Fig. 1d). This method eliminated lengthy high magnification image acquisition on the entire microscope glass, and facilitated fast zoom-in to the optimal focusing plane. To normalize vignetting optical signals across the image of the whole tissue slice prior to image analysis, a montage image of the ROI was captured with 10% overlap between field-of-views (FOV) for subsequent image analysis processing (Fig. 1d). An advanced flat-field correction function built-in Harmony software was also applied to all FOVs. With this approach, it took 30 min to complete a 4-color image acquisition for one 1 cm × 1 cm tissue area, at least 6 times faster than a standard tissue slide scanner in its fluorescent mode.

### Development of DL-based image analysis

**Figure 2:**
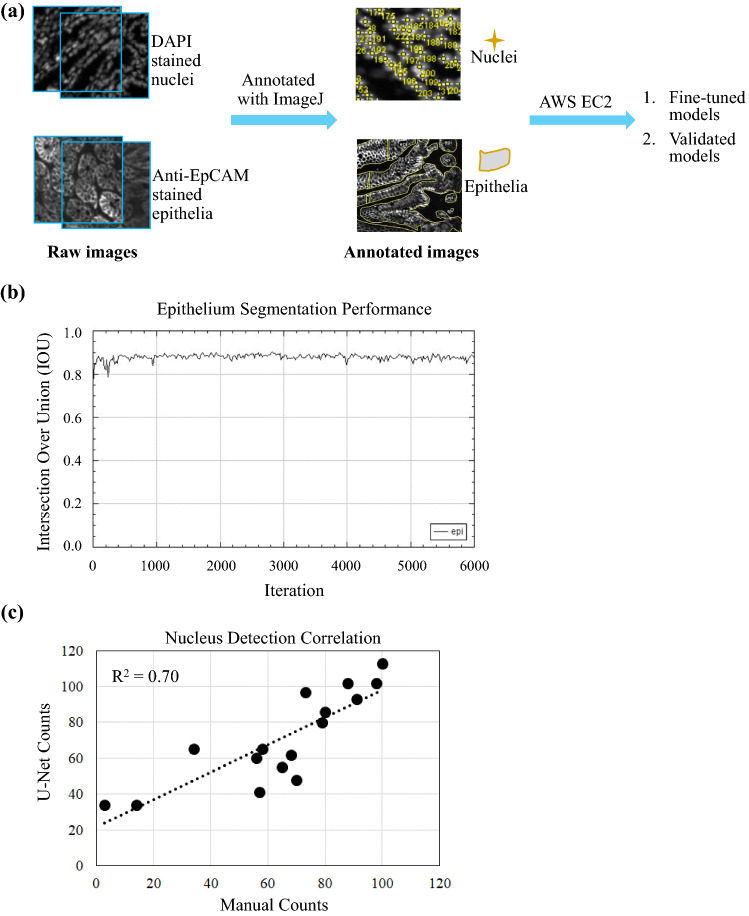
U-Net based image analysis workflow. (**a**) Raw images of colon or small intestine with DAPI stained nuclei and anti-EpCAM stained epithelial were annotated separately by biologists using ImageJ. Annotated images were used to fine-tune or to validate U-Net models residing in AWS EC2. (**b**) The performance of epithelium segmentation was evaluated with Intersection Over Union (IOU). (**c**) The performance of nucleus detection was assessed by correlating U-Net counting with manual counting across 16 small tissue regions.

Transfer learning was performed to repurpose U-Net, an open source DL model, for HCS-based tissue imaging analysis. In order to develop a scalable imaging annotation process, ImageJ was adapted as an enabling tool for biologists to handcraft the features-of-interest. Beside auto contrast, no other special treatment was performed on the images prior to manual depiction on features-of-interest. Manual image annotation was conducted using ImageJ located on a desktop computer. Guided by a pathologist, biologists first selected slides with the most abundant phenotypes on typical colon or intestine cellular and tissue structures, then annotated these slides using two methods. First, segmentation of epithelial regions was achieved by using “Freehand Selection” function in ImageJ to outline the EpCAM stained epithelial areas. Second, each nucleus was identified by point clicking DAPI stained nuclei using the “Multi-Point” function. Facilitated by a U-Net ImageJ plug-in, annotated images were loaded to U-Net models^12,16^ hosted on an Amazon Web Services Elastic Compute Cloud (AWS EC2) instance with GPU. These images were used for model training or validation (Fig. 2a). One U-Net model was fine-tuned and validated to segment epithelial regions with EpCAM staining, another model was attuned and validated to detect nuclei stained with DAPI. For the model on epithelium segmentation, 9 colon images (3 each from distal, medial and proximal sections of colon) were used for fine-tuning the model, and 4 images each from colon (2 proximal, 1 distal and 1 medial sections) and small intestine (2 duodenum, 1 jejunum and 1 ileum sections) were used for testing and validation. With 6,000 iterations and a learning rate at 10^–5^, the model achieved an acceptable performance on the validation set. The resulting Intersection-Over-Union (IOU)^12^ score was 0.88 (Fig. 2b) for colon and 0.85 for small intestine. IOU is a commonly used metric to quantify the performance of machine learning and deep-learning models on object segmentation. An IOU value of 1 indicates a perfect model, and greater than 0.8 is broadly considered as a high quality model. For the model on nuclei counting, 5 colon images (2 proximal, 2 distal and 1 medial sections) were used for fine-tuning and 1 image each from colon (proximal section) and small intestine (duodenum section) were used for testing and validation. With 10,000 iterations and a learning rate at 10^–5^, the model also achieved a satisfactory performance on the validation image. The manual and U-Net counts on nuclei across 16 regions of the validation image set were highly correlated (R^2^ = 0.70, P < 0.0001) (Fig. 2c). Overall, it is determined that the DL-based nuclear detection and epithelial region segmentation was about 500 times faster than manual operation.

### Application of DL models for quantifying drug biodistribution

**Figure 3:**
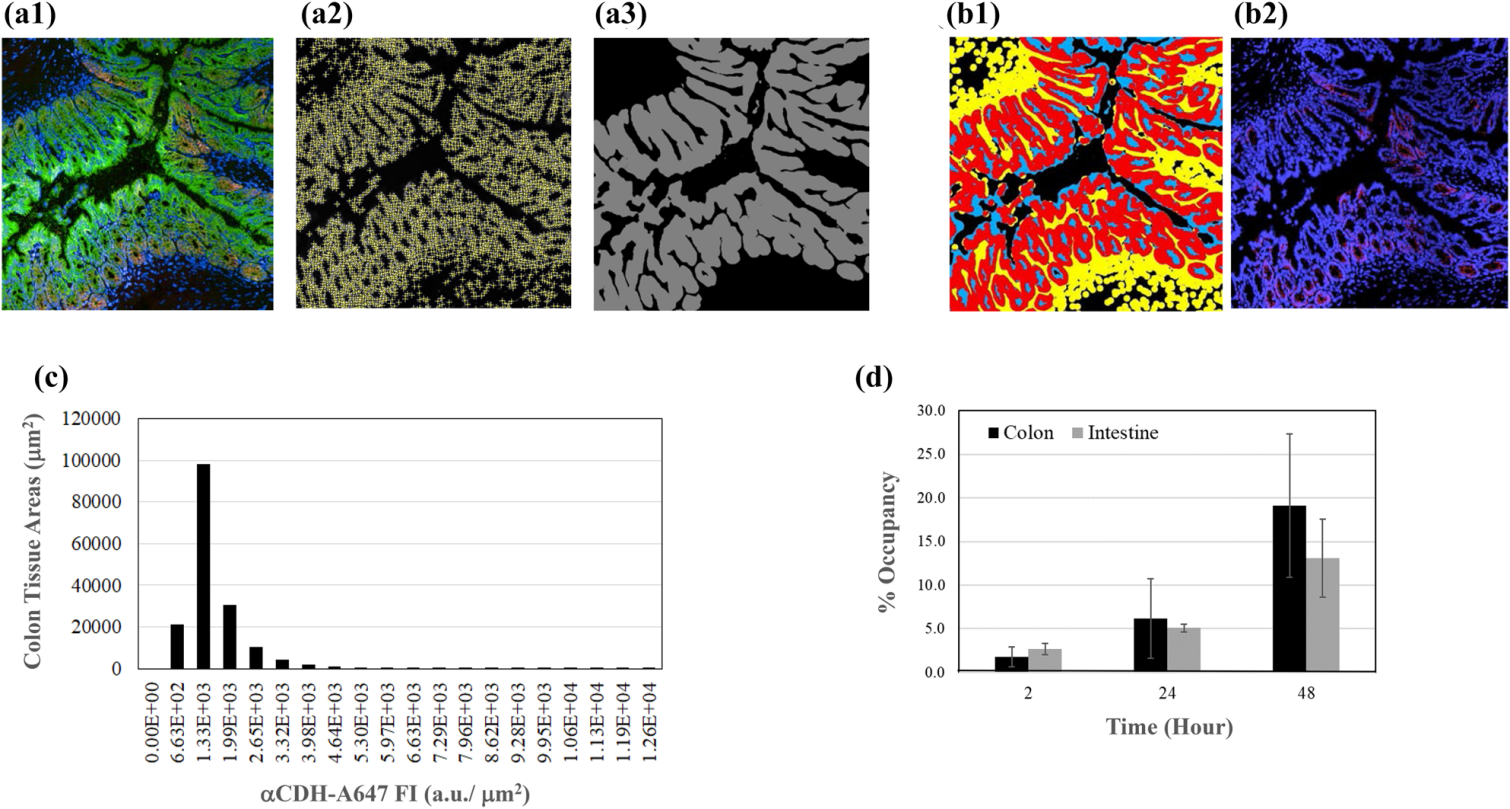
Example of applying U-Net models to determine drug biodistribution in production run. (**a**–**c**), a FOV on proximal section of colon tissue obtained from mouse dosed with 3 mg/Kg αCDH-A647 for 24 h. (**a1**) Full color image of the FOV acquired by HCS (red: αCDH-A647; green anti-EpCAM; blue: DAPI). (**a2**) Nuclei identified by a U-Net model (yellow cross). (**a3**) Epithelial region segmented by another U-Net model (grey color zone). (**b1**) Identification of nucleated epithelial region in the FOV—yellow: nuclear areas approximated from detected nuclei; blue: segmented epithelial regions; red: selected nucleated epithelial regions by overlapping yellow and blue. (**b2**) Image of αCDH-A647 (red) with nuclei (blue) in the FOV. (**c**) Histogram of αCDH-A647 biodistribution in the FOV was obtained by overlapping αCDH-A647 in (**b2**) with nucleated epithelial regions in (**b1**). (**d**) Time courses of αCDH-A647 occupancy in nucleated epithelial cells of the whole colon proximal and small intestine duodenum tissue sections.

To apply the fine-tuned model for a production run analysis, the biologists opened new images in ImageJ and chose the model in the plug-in along with appropriate parameters. The plug-in then guided the images, the model name and parameters to the AWS EC2 host for model inference (analysis and result extrapolation). The outputs subsequently were presented by the plug-in as images and/or coordinates of the detected structures (Fig. 3a1–a3). After identification of nucleus and epithelial areas, nucleated epithelial regions were determined by dilating 7 μm in radius from the identified nucleus to the approximate size^18^ of an entire cell (Fig. 3b1). αCDH-A647 that co-localized with these nucleated epithelial regions was considered as specific on-target binding that would lead to desirable therapeutic efficacy and the rest of αCDH-A647 were non-specific binding (Fig. 3b2). The corresponding αCDH-A647 distribution in the nucleated epithelial regions per μm^2^ was visualized by a histogram (Fig. 3c). Finally, the time courses of αCDH-A647 occupancy were determined in whole colon proximal and small intestine duodenum tissue sections (Figs. 3d, S1 and S3). Although the time courses and degrees of αCDH-A647 occupancy were very similar between whole colon and small intestine tissue slices, within each tissue type, αCDH-A647’s distribution was highly heterogeneous (Figs. S1, S2).

## Discussion

This quantitative and high throughput tissue image analysis method can be generalized for drug distribution studies in different tissue types in animal models. The high throughput and whole tissue slice imaging technology allows one to appreciate the heterogeneity of drug binding within selected organs from different animals. Coupling with multiplexicity and superior resolution provided by confocal HCS technology, the deep learning nature of image analysis tolerates some degree of blurriness associated with frozen tissue images as well as variations from different FOVs, allows exclusion of non-specifically trapped drug in interstitial spaces, and more importantly, enables differentiation of drug binding to desirable and undesirable cell types.

Standard technologies, such as total fluorescent (Fig. S3) or radiography-based bioimaging^8^ methods could provide ROI-level spatial resolutions for drug biodistribution studies. However, due to image resolution limitations, these technologies cannot provide cell-level spatial resolution needed for discriminating different drug binding modes and associated therapeutic mechanism of actions. The technology described in this manuscript provides significant advantages over the standard technologies by enabling cell-level quantification, thus more accurate interpretation of drug binding course. The multiplexed capability of HCS would allow co-detection of downstream cellular events, such as target engagement or pharmacodynamic biomarkers. Together with more precise assessment of drug binding, this integrated technology would enable one to more accurately correlate drug binding with therapeutic efficacy or adverse effects.

## Methods

### Slide preparation

The anti-CDH17 antibody was raised in house and fully validated. It was labeled with Alexa Fluor 647 dye using succinimidyl ester method following manufacturer’s instruction (ThermoFisher, catalog # A20006). The Alexa Fluor 647 conjugated anti-CDH17 antibody (αCDH-A647, antibody to fluorophore ratio = 1:4) was injected into mice at 3 mg/Kg concentration, which was pre-selected based on balances between efficacy and toxicity. Colon and small intestine tissues were collected from different mice treated with αCDH-A647 for 2, 24 and 48 h. The tissues were then frozen, and sectioned to 5 μm slices prior to staining. A FITC conjugated EpCAM antibody (ThermoFisher, catalog # 11-5791-82) was used to detect epithelial cells in the colon and small intestine. Nuclei were labeled with DAPI.

### Image acquisition

Image acquisition of the microscope slide was conducted on the Opera PHENIX™ HCS imager (PerkinElmer, Waltham, MA) equipped with laser microlens confocal and a large 4.7 Mpixel CMOS camera. Fluorescent dyes used for labeling tissues were matched with appropriate laser excitation light sources and complementary emission filters [Nucleus (DAPI): ex 375 nm, em 435–480 nm; drug (αCDH-A647): ex 640 nm, em 650–760 nm; epithelial cells (EpCAM-FITC): ex 488 nm, em 500–550 nm]. To increase image acquisition throughput, a slide holder housing four slides per run was used (Fig. 1a, b). The accompanying image acquisition and image analysis software called Harmony 4.9 (PerkinElmer, Waltham, MA) was used to streamline the image capture process. PRECISCAN, a method built-in Harmony 4.9, was used to pre-scan and re-scan ROI automatically. The software first performed a low magnification (air objective lens 5 ×, NA = 0.16) tissue topography survey (x, y & z locations) based on all nuclear stains to define ROI. Based on this preliminary survey, a second round of multi-color image acquisition on the ROI was performed at a higher magnification (water objective lens 20X, NA = 1.0). This method prevented lengthy image acquisition on the entire microscope slide, and facilitated the identification of the optimum focus plane. To minimize vignetting optical signals across the whole tissue slice, a montage image of the ROI was captured with 10% overlap between FOVs for subsequent image analysis (Fig. 1d). An advanced flat-field correction function built-in Harmony 4.9 was also applied to all FOVs.

### Image analysis

We installed the U-Net package on an AWS EC2 according to https://lmb.informatik.uni-freiburg.de/resources/opensource/unet/. The package was constructed in Caffe deep-learning framework (https://arxiv.org/abs/1408.5093), using weighted soft-max cross-entropy as the loss function to optimize the network parameters^12^. It included a model trained with 2-dimentional tissue images of various modalities, 2D Cell Net (v0), from which our epithelial and nuclear models were fine-tuned with annotated images acquired by HCS. As previously described^12^, fine-tuning, validation and application of the U-Net model were facilitated with a GUI provided by an ImageJ plug-in so that a user without coding experience can carry out the entire process. For training, the user opened a group of annotated images in ImageJ, and assigned them as training or validating image sets in the plug-in. The plug-in then sent the image sets along with user-provided parameters to the AWS EC2 where the pre-trained models were fine-tuned and validated. To apply a fine-tuned model for production run analysis, the user opened a new image in ImageJ and chose the model in the plug-in along with appropriate parameters. The plug-in then sent the images, the model names and parameters to the EC2 host for inference. The outputs of the inference were presented by the plug-in as images and/or coordinates of the detected structures. Performance of epithelial segmentation was measured by the Intersection Over Union (IOU)^12,19^, the intersection of predicted segmentation and ground-truth annotation divided by their union. To compare manual and U-Net detection of nuclei, the annotated region in the testing image was divided into 16 squares of 104 microns across. The pair of manual and U-Net counts of nuclei in each square was plotted to calculate R^2^ value.

### Mice and ethics statement

The animal studies were approved by the Bristol-Myers Squibb Animal Care and Use Committee, and all methods were performed in accordance with National Institutes of Health Guide for the Care and Use of Laboratory Animals (National Academies Press, Washington, DC, 8th Ed.).

## Supplementary information

Supplementary information
