# Supplementary material for: Quantifying drug tissue biodistribution by integrating high content screening with deep-learning analysis
Source: Sci Rep. 2020 Sep 1;10:14408. doi: 10.1038/s41598-020-71347-6 (PMC7463244; doi:10.1038/s41598-020-71347-6)
Supplement: Supplementary file 1 — Supplementary information [file 41598_2020_71347_MOESM1_ESM.pdf]

Quantifying Drug Tissue Biodistribution
by Integrating High Content Screening with Deep-Learning Analysis

Zhuyin Li[1]*⊥, Youping Xiao[2⊥], Jia Peng[1], Darren Locke[3], Derek Holmes[4], Lei Li[1], Shannon Hamilton[1], Erica Cook[1], Larnie Myer[1], Dana Vanderwall[2], Normand Cloutier[2], Akbar M. Siddiqui[2], Paul Whitehead[2], Richard Bishop[2], Lei Zhao[5] and Mary Ellen Cvijic[1]

[1]Lead Discovery and Optimization, Bristol-Myers Squibb, Princeton, NJ. [2]Information Technology for R&D, Bristol-Myers Squibb, Princeton, NJ. [3]Translational Medicine, Bristol-Myers Squibb, Princeton, NJ. [4]Immunoscience Biology Discovery, Bristol-Myers Squibb, Princeton, NJ. [5]Cardiovascular Translational Research, Bristol-Myers Squibb, Hopewell, NJ.

*Corresponding Author:
Zhuyin Li
Bristol-Myers Squibb
3551 Lawrenceville Road, Princeton, NJ 08540
Phone: 609-252-7681
Zhuyin.Li@bms.com

⊥These two authors contributed equally.

Figure S1. Full color original image of colon proximal section acquired by HCS, red: αCDH-A647; green anti-EpCAM; blue: DAPI. 24 hours after treatment.

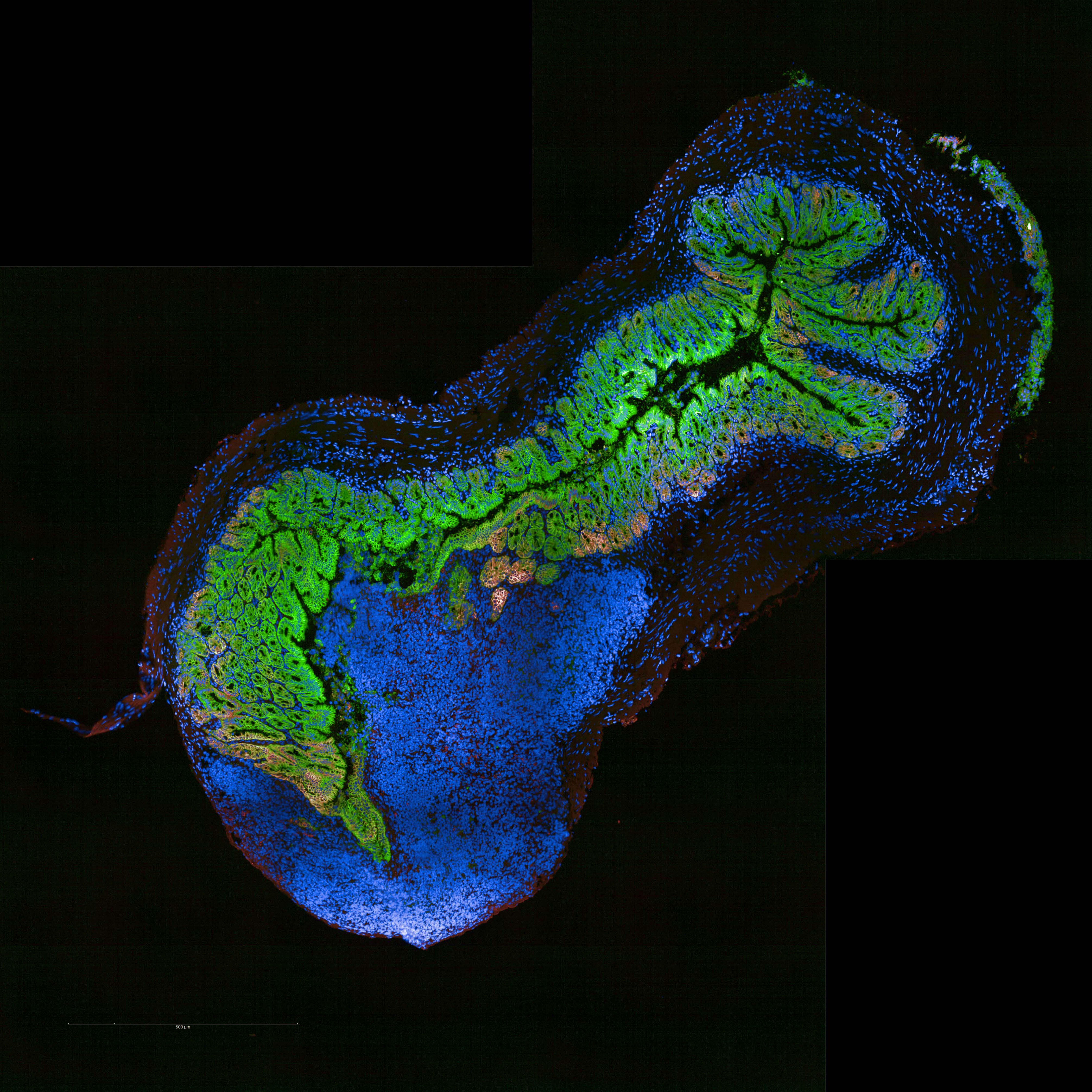
500 µm

Figure S2. Full color original image of small intestine duodenum section acquired by HCS, red: αCDH-A647; green anti-EpCAM; blue: DAPI. 24 hours after treatment.

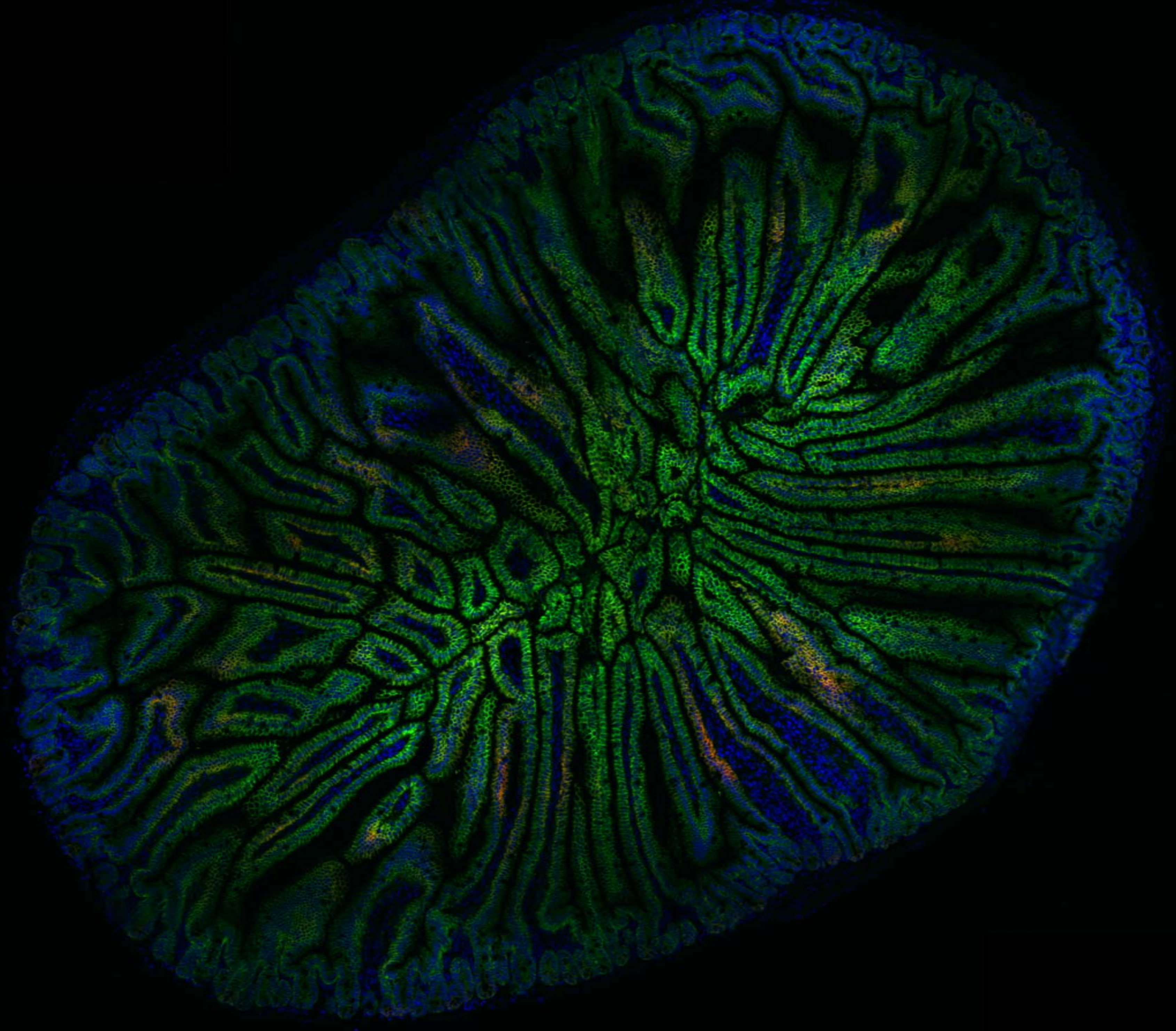

Figure S3. Time courses of drug binding to proximal sections of colon and duodenum sections of small intestine determined by total fluorescent intensity of αCDH-A647. The same tissue sections used in Figure 3d. This quantification method cannot separate non-specific binding from specific binding, which led to wrong interpretation of drug binding time course.

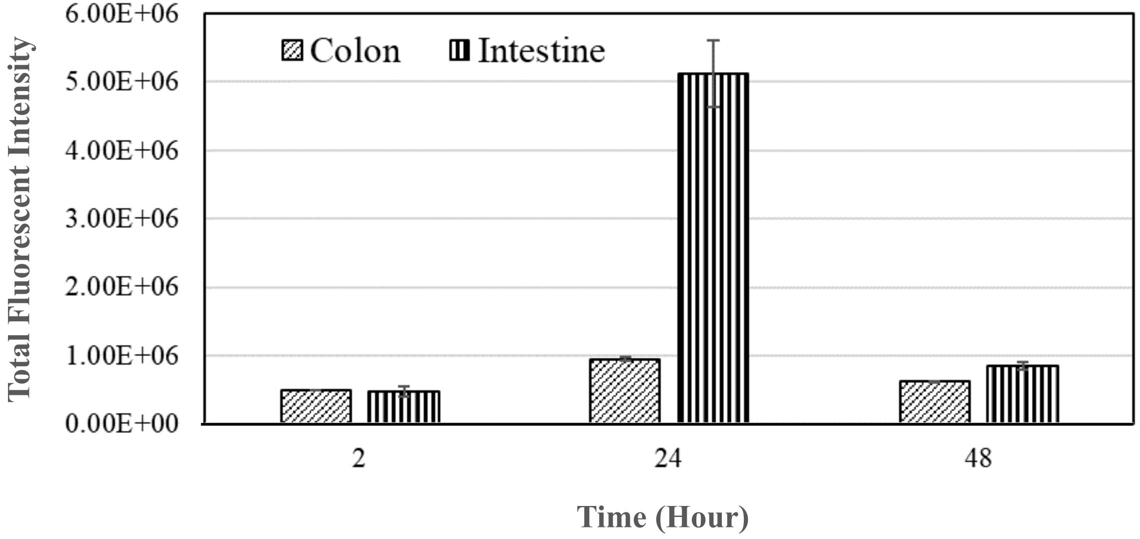

Colon
Intestine
Total Fluorescent Intensity
6.00E+06
5.00E+06
4.00E+06
3.00E+06
2.00E+06
1.00E+06
0.00E+00
2
24
48
Time (Hour)
